# Unveiling the Physiological Basis of Cold Tolerance in Maize: Root Architecture, Photosynthetic Stability, and POD-Mediated Defense Under Delayed Chilling Stress

**DOI:** 10.3390/plants15030517

**Published:** 2026-02-06

**Authors:** Zhen Wang, Qi Jia, Baolin Zhang, Bo Ming, Lanfang Bai, Fugui Wang, Yongqiang Wang, Shengnan Yu, Runhou Zou, Zhigang Wang

**Affiliations:** 1 College of Agronomy, Inner Mongolia Agricultural University, Hohhot 010019, China; cau1022@imau.edu.cn (Z.W.); lfbai@imau.edu.cn (L.B.); fgwang2008@163.com (F.W.); wangyongqiang@nwafu.edu.cn (Y.W.); imauyusn@163.com (S.Y.); imauhou@163.com (R.Z.); 2 Inner Mongolia Autonomous Region Industrial Technology Engineering Center for Intelligent Water and Fertilizer Management Technology and Equipment for Crops, Hohhot 010019, China; 3 Chifeng Bureau of Agriculture and Animal Husbandry, Chifeng 024000, China; imaujiaq@163.com; 4 College of Chemistry and Environmental Sciences, Inner Mongolia Normal University, Hohhot 010020, China; zhangbl@imnu.edu.cn; 5 Institute of Crop Sciences, Chinese Academy of Agricultural Sciences, Beijing 100081, China; mingbo@caas.cn

**Keywords:** chilling stress, maize (*Zea mays* L.), antioxidant system, root morphology, photosynthesis

## Abstract

Delayed chilling stress is a frequent meteorological disaster in the spring maize-growing region of Northern China. Understanding the physiological responses and key characteristics of cold-tolerant maize varieties under such stress is crucial for their selection and utilization. This study compared the physiological and biochemical responses of a cold-tolerant variety (XY335) and a conventional variety (KH8) to simulated delayed chilling stress induced by early field sowing. Results showed that the emergence percentage and emergence uniformity of the cold-tolerant variety were 9.6% and 2.8% higher than those of the conventional variety, respectively. Under chilling stress, the root diameter of the cold-tolerant variety remained stable, while root length decreased by 24.5%. In contrast, the conventional variety exhibited the opposite response. Growth of the cold-tolerant variety slowed during stress but accelerated significantly after temperature recovery, achieving 1–2 more leaf ages than the conventional variety. The SPAD value (chlorophyll content) of the cold-tolerant variety was less affected, remaining 14.3% higher than the conventional variety, thereby maintaining higher photosynthetic efficiency. The enhanced stress tolerance of XY335 correlated with a robust antioxidant system: leaf peroxidase (POD) activity was 60.7% higher, and malondialdehyde (MDA) content was 42.4% lower compared to KH8. In summary, under delayed chilling stress, the cold-tolerant variety ensured higher emergence and seedling uniformity by reducing coleoptile length, maintained root diameter and absorption capacity by shortening root length, preserved chlorophyll synthesis and photosynthetic performance under the protection of a POD-dominated enzyme system, and employed a “standby mode” with compensatory leaf growth to ensure adequate dry matter accumulation and yield formation.

## 1. Introduction

Climate instability has increased significantly over the past 30 years. Fluctuations in first and last frost dates have intensified, and the frequency of meteorological disasters like chilling stress and cold damage has risen markedly [1]. Chilling stress, a natural disaster occurring when low non-freezing temperatures impact crop growth, detrimentally affects development and reduces yield [2]. It encompasses delayed chilling stress, obstructive chilling stress, and mixed chilling stress. In the spring maize-growing region of northern China, delayed chilling stress is a major meteorological disaster, severely impacting maize nutrition, growth, maturation, yield, and grain quality. Selecting cold-tolerant varieties is vital for stabilizing maize production. Therefore, elucidating the physiological mechanisms and identifying key characteristics of cold tolerance in maize varieties is essential for their effective selection, utilization, and cultivation.

Delayed chilling stress primarily affects the vegetative growth stage, such as during seed germination and seedling emergence. Low temperatures weaken plant growth and metabolism, leading to delayed phenology, failure to mature on time, and yield reduction [3]. Previous studies indicate temperatures as low as 6 °C before seedling emergence can cause seed/seedling death, delayed emergence, and reduced seedling vigor [3]. Low temperature significantly influences plant height, fresh weight, leaf age, and relative growth rate. It decreases root cap cell proliferation and absorption activity, reduces the root-to-shoot ratio and root dry weight, ultimately impairing water and nutrient uptake in maize seedlings [4]. Inhibition of root growth indirectly suppresses leaf growth. During the seedling stage, stem and leaf development are vulnerable to low temperatures, manifesting as reduced plant height, leaf wilting or water-soaked lesions, stem apex browning or yellowing, and even local necrosis or death [2]. Chilling stress primarily inhibits plant growth, a process that may involve constraints in cell-to-cell communication and metabolism. This growth inhibition is often accompanied by an accumulation of reactive oxygen species (ROS), which can disrupt cellular redox homeostasis. Under prolonged or severe stress, ROS may further lead to oxidative damage to cellular components, exacerbating dysfunction [5]. Under chilling stress, maize seedling survival depends on their adaptive capacity, involving increased synthesis and activity of antioxidant enzymes like superoxide dismutase (SOD), catalase (CAT), and peroxidases (PODs) [5]. However, the primary or most critical antioxidant enzymes involved remain unclear.

Therefore, understanding the biochemical and physiological basis of maize chilling tolerance is crucial for developing strategies to mitigate its effects. This study compared a cold-tolerant variety and a conventional variety under simulated delayed chilling stress via early sowing. It aimed to provide an integrated physiological profile of chilling tolerance by simultaneously examining key traits—from germination and root architecture to photosynthesis and the antioxidant system—under field-simulated conditions. We specifically sought to (i) identify which components of the antioxidant defense are most strongly associated with tolerance, (ii) characterize the growth and resource allocation strategies that enable tolerant varieties to maintain yield stability, and ultimately (iii) elucidate the integrated physiological basis of chilling tolerance to inform the establishment of a practical index system for variety selection.

## 2. Results

### 2.1. Degree of Delayed Chilling Stress During the Maize Growth Period

The relative active accumulated temperature anomaly from emergence to V7 was −2.1% (2017) and −2.5% (2018). These negative TXJ values confirm a significant active temperature deficit during this early growth period, i.e., the occurrence of delayed chilling stress. Based on the threshold values (Table 1), this deficit corresponded to severe delayed chilling stress in both years, with higher intensity in 2018.

### 2.2. Effect of Delayed Chilling Stress on Maize Germination

Chilling stress prolonged germination time and reduced germination percentage (Table 2). Under NT, germination percentage did not differ significantly between varieties. Under LT, XY335 germination percentage was 39.1% higher than that of KH8. XY335’s GI was 127.3% higher than KH8 under LT. SVI decreased significantly under LT, but XY335’s SVI remained 100% higher than KH8. Coleoptile length decreased significantly under LT (XY335: −72.8%; KH8: −67.2%). While no difference existed under NT, KH8 coleoptile length was 16.2% longer than XY335 under LT, indicating XY335 maintained germination by shortening coleoptile length.

### 2.3. Effect of Delayed Chilling Stress on Emergence of Maize

Chilling stress decreased emergence percentage, emergence index, and seedling uniformity, with smaller reductions in XY335 compared to KH8 (Table 3). In 2017, the XY335 emergence percentage, EI, and SU were 10.0%, 4.3%, and 10.7% higher than those of KH8 under CS. In 2018, XY335 emergence percentage and EI were 9.3% and 11.1% higher than KH8 under CS, though SU was 6.2% lower (no significant difference indicated in the table; note: the table shows values, but significance letters are key). Averaged over years, XY335 emergence percentage, EI, and SU under CS were 9.6%, 7.3%, and 2.8% higher than KH8, respectively.

### 2.4. Effect of Delayed Chilling Stress on Maize Root Growth

**Figure 1:**
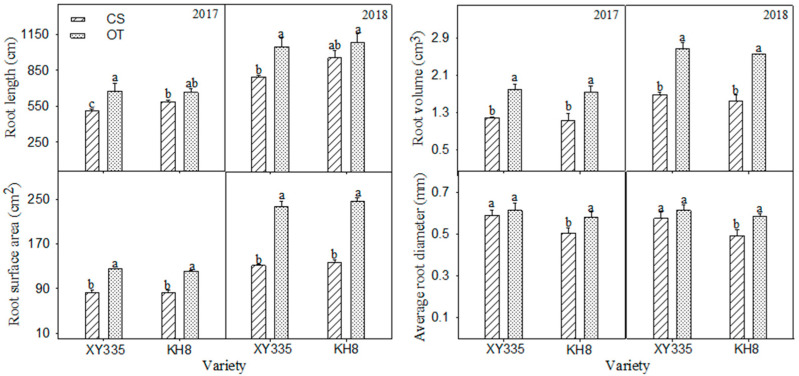
Response of root growth parameters of different cold-tolerance maize varieties to chilling stress (CS vs. OT). Bars with different letters within a parameter differ significantly (LSD, *p* < 0.05). Error bars represent the standard error of the mean. (Root length refers to total root length per plant within the 0–30 cm soil layer).

Under CS, no significant differences existed in root length, volume, or surface area between varieties, but XY335 had a significantly higher average root diameter than KH8 (Figure 1). CS significantly decreased root length, volume, and surface area in XY335, but not average diameter. In KH8, CS significantly decreased root volume, surface area, and diameter, but not root length. The decline in root diameter was significantly smaller in XY335 (−8.3%) than in KH8 (−12.8%). Thus, the cold-tolerant variety maintained root diameter by reducing root length under stress.

### 2.5. Effect of Delayed Chilling Stress on Maize Leaf Growth

**Figure 2:**
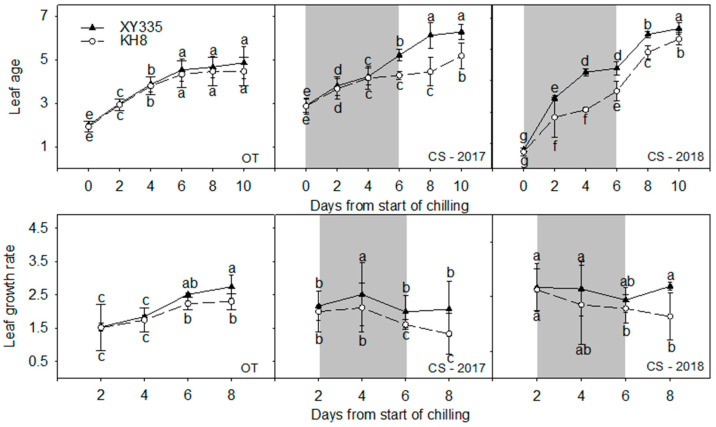
Leaf growth (leaf age) of different maize varieties under chilling stress (CS) and non-stress (OT) conditions. Shading indicates the chilling stress period. Bars/dots with different letters within a measurement day differ significantly (LSD, *p* < 0.05). Error bars represent SE.

Chilling stress significantly decelerated leaf growth in both varieties, with a more severe effect on KH8 (Figure 2). XY335 maintained a relatively stable leaf growth rate during stress, which increased rapidly after temperature recovery. KH8’s growth rate decreased progressively during stress and remained significantly lower than that of XY335. Ten days after stress onset, XY335 exhibited 1–2 more leaf ages than KH8.

### 2.6. Effects of Delayed Chilling Stress on Leaf Photosynthesis of Maize

**Figure 3:**
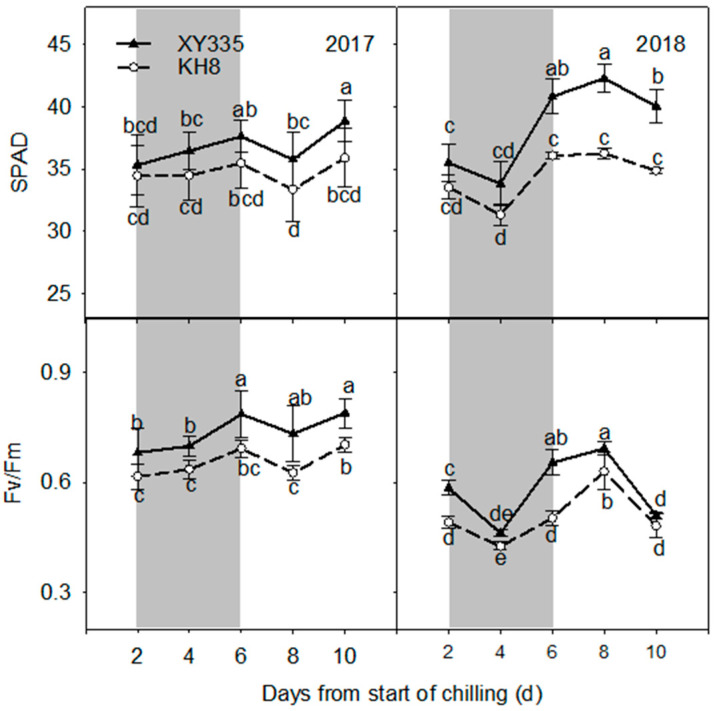
Comparison of SPAD and Fv/Fm values in leaves of different maize varieties under chilling stress. Shading indicates the chilling stress period. Bars/dots with different letters within a measurement day differ significantly (LSD, *p* < 0.05). Error bars represent SE.

Chilling stress inhibited chlorophyll synthesis in KH8 but not significantly in XY335 (Figure 3). XY335’s SPAD value was significantly higher than KH8’s, and the difference widened with prolonged stress. This inhibition indirectly suppressed photosynthetic capacity. In both years, the maximum photochemical efficiency (Fv/Fm) of PSII in KH8 remained low during stress. In contrast, XY335 showed an increasing trend and was significantly higher than KH8, indicating less inhibition of PSII potential activity.

**Figure 4:**
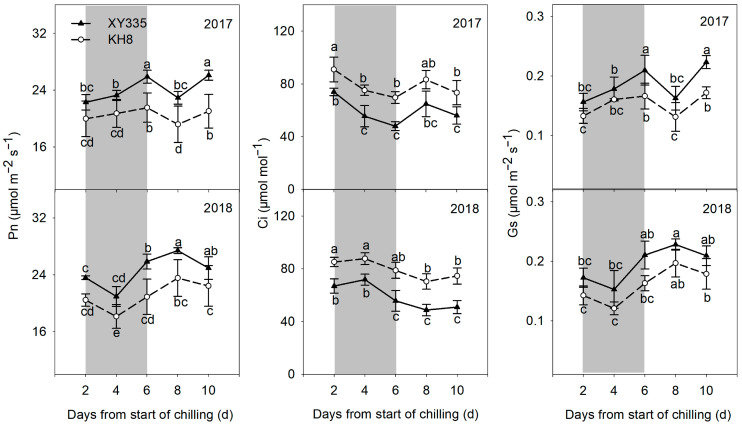
Comparison of leaf photosynthetic parameters (Pn, Ci, and Gs) of different maize varieties under chilling stress. Shading indicates the chilling stress period. Bars/dots with different letters within a measurement day differ significantly (LSD, *p* < 0.05). Error bars represent SE.

The photosynthetic rate (Pn) of KH8 was significantly lower than that of XY335 under CS (Figure 4). However, intercellular CO_2_ concentration (Ci) was significantly higher in KH8, indicating impaired CO_2_ fixation. Stomatal conductance (Gs) did not differ significantly between varieties under stress, suggesting photosynthetic inhibition was primarily non-stomatal.

### 2.7. Effects of Delayed Chilling Stress on Antioxidant Defense System of Maize

**Figure 5:**
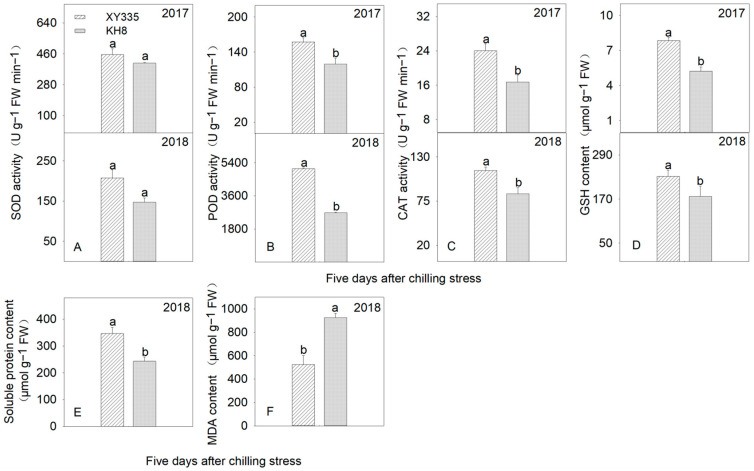
Comparison of antioxidant enzyme activities (POD, total superoxide dismutase (SOD) activity, and CAT—(**A**–**C**)), non-enzymatic antioxidants (GSH and Pro—(**D**,**E**)), and membrane damage (MDA—(**F**)) in leaves of different maize varieties under chilling stress. Bars with different letters within a parameter differ significantly (independent sample *t*-test, *p* < 0.05). Error bars represent SE.

After 5 days of CS, the antioxidant protection system (enzymatic and non-enzymatic) of XY335 was significantly more active than that of KH8 (Figure 5). For enzymatic systems (2-year average), POD, SOD, and CAT activities in XY335 were 60.7%, 26.6%, and 39.1% higher than KH8, respectively. For non-enzymatic systems, GSH content in XY335 was 50.3% (2017) and 30.8% (2018) higher than in KH8. Proline (Pro) content, a major osmoprotectant, was 42.6% higher in XY335 leaves.

MDA content, reflecting membrane lipid peroxidation, was 42.4% lower in XY335 than in KH8, demonstrating the effectiveness of its antioxidant and osmotic regulation systems in reducing membrane damage under stress (Figure 5F). The highest negative correlation coefficient was found between MDA content and POD activity (−0.976, *p* < 0.001), highlighting the particular importance of POD activity within the antioxidant system under chilling stress.

### 2.8. Effects of Delayed Chilling Stress on Dry Matter Accumulation and Yield of Maize

The impact of chilling stress on emergence and seedling growth ultimately affected dry matter accumulation and yield (Table 4). Biomass of XY335 at R1 did not decrease significantly under CS, whereas KH8 biomass decreased significantly by 14.4% (2017) and 15.0% (2018). XY335 yield reduction under CS was not significant, while KH8 yield per plant and grain yield decreased by 10.7% and 6.8% in 2017 and 2018, respectively. This indicates the cold-tolerant variety maintained higher dry matter accumulation under stress, leading to better yield stability.

## 3. Discussion

### 3.1. Germination and Seedling Establishment Under Chilling Stress

The process of seed germination is highly sensitive to temperature. While chilling tolerance in maize has been studied from various angles, our integrated approach under simulated field conditions reveals how tolerant varieties coordinate distinct physiological adaptations across different organizational levels. Delayed chilling stress in northern Chinese springs often inhibits maize germination and seedling growth. Emergence ability is a key indicator of maize cold tolerance [6,7]. This phenotypic advantage is supported by genetic studies; for instance, recent QTL and transcriptomic analyses have identified specific genes (e.g., ZmbZIP113 and ZmTSAH1) that positively regulate low-temperature germination ability in maize, highlighting the genetic underpinnings of this critical trait [8]. Novel approaches, such as pre-treatment with a high-voltage electrostatic field (HVEF), have been shown to significantly enhance seed vigor, soluble sugar accumulation, and subsequent seedling growth under chilling stress by improving cell wall properties and osmotic regulation [9]. Our findings confirm that cold-tolerant varieties emerge faster and more uniformly under stress, forming a critical foundation for yield.

### 3.2. Root Architectural Plasticity as an Adaptive Trait

While cold tolerance selection at the seedling stage often focuses on root/seedling weight [10,11], systematic root morphology studies are limited. Hund et al. [12] proposed that chilling stress primarily inhibits maize growth via low soil temperature effects on roots. Temperatures below 15 °C reduce root dry weight and alter root tip morphology [13]. Recent studies have highlighted that root hair development and overall root system architecture are highly sensitive to specific cold stress intensities, with severe cold triggering distinct transcriptomic reprogramming related to cell wall remodeling and hormone signaling [1].

Our study confirms that CS significantly reduces root surface area and volume. Crucially, the cold-tolerant variety adapted by shortening root length to maintain root diameter, ensuring higher seedling vigor, growth, and ultimately better emergence traits than the sensitive variety. This suggests root length and diameter plasticity are key morphological differences defining chilling tolerance, with a shift towards a compact, robust root system being a key adaptive feature that aligns with the “stress avoidance by architectural modulation” strategy observed in other cereals under abiotic stress [14]. This morphological adaptation is part of a broader cold acclimation strategy that includes remodeling of membrane lipids to maintain fluidity and function under low temperatures [15]. This root morphological plasticity in tolerant varieties likely enhances soil contact volume and root vigor, improving nutrient capture efficiency [16]. Reduced root hydraulic conductivity under CS also indirectly affects leaf water status, potentially causing stomatal abnormalities [17]. Soil temperature influences shoot meristem development before the six-leaf stage, as the shoot tip remains belowground [18].

### 3.3. Leaf Growth Dynamics and the “Standby Mode”

Our study showed that CS delayed leaf growth by reducing the emergence rate. XY335 maintained a higher leaf growth rate during stress and recovered faster afterward. Riva-Roveda et al. [19] observed European flint corn varieties entering a “standby mode” at low temperatures. Similarly, XY335 exhibited arrested leaf growth during stress but rapid compensatory growth upon warming, indicating that a “standby mode” is another key feature of cold tolerance. This phenomenon is corroborated by kinematic analyses showing that the cellular basis of recovery (cell division and elongation rates) differs significantly depending on whether leaves are chilled before or after emergence, with emerged leaves showing faster and more complete recovery [20]. This capacity to reversibly pause and restart growth may be linked to the dynamic regulation of cell cycle genes and metabolic reprogramming towards cold acclimation, a conserved response mechanism emerging in plant stress biology [21].

### 3.4. Photosynthetic Stability and Chloroplast Function

Chilling stress could lead to many physiological disorders in plants [22]. In this study, we observed that chilling stress decreased leaf chlorophyll content, suggesting that chlorophyll synthesis of maize leaves was obstructed by chilling stress. Compared to the cold-sensitive variety, the cold-tolerant variety retained a higher chlorophyll content during chilling stress. Low temperature has been shown to affect leaf photosynthesis, including water and gas exchange, photosynthetic electron transfer, and carbon assimilation, and seriously affect the formation of photosynthetic products [23,24,25]. The main factors that reduce photosynthetic capacity under adverse conditions include stomatal and nonstomatal restriction; stomatal restriction is caused by the partial closure of stomata, while nonstomatal restriction is caused by the decline in photosynthetic activity of mesophyll cells [26]. Kao et al. [27] found that the net photosynthetic rate and stomatal conductance of mangrove were reduced after being treated at low temperature for one hour, and noted that the reason for the decrease in light saturation rate was stomatal closure induced by the low temperature. Similarly, Yang [28] noted that the decrease in net photosynthetic rate was closely related to the decrease in stomatal conductance at 5 °C during a 13 h night. In this study, Pn and Gs of maize leaves decreased under chilling stress, but Ci increased significantly. The decrease in stomatal conductance caused by low temperature did not reduce the intercellular CO_2_ concentration, which indicated that the increase in Ci was unrelated to stomatal factors. Therefore, the main reason for photosynthetic changes in maize leaves at low temperature was nonstomatal restriction, as found by Han et al. [29] under low-temperature stress. These results show that the nonstomatal limitation may be due to changes in CO_2_ fixation capacity, photosynthetic pigment content, key photosynthetic enzymes, and other factors, which indirectly resulted in reduced photosynthetic activity of mesophyll cells and decreased Rubisco content [30]. The present study found that Pn and Gs of the cold-tolerant variety were higher than those of the cold-sensitive variety, but Ci concentration was significantly lower than that of the cold-sensitive variety. These results indicated that the CO_2_ fixation ability of the cold-sensitive variety decreased significantly under chilling stress, and the reduction in carbon assimilation ability further reduced assimilatory power and the intermediate metabolites produced by the light reactions, leading to decreased carbon and nitrogen metabolism, and affected a series of physiological and biochemical activities related to metabolism. The cold-tolerant variety maintained a higher photosynthetic carbon assimilation ability, stable carbon and nitrogen metabolism, and thus, maintained its chlorophyll content under chilling stress, providing an important basis for light energy absorption and electron transfer. This phenomenon constitutes a virtuous cycle in the photosynthetic system of the cold-tolerant variety under chilling stress, and finally ensures maize yield and quality under low-temperature conditions.

The effects of chilling stress on photosynthetic performance of maize seedlings include the effects on photosynthetic chemical reactions, PSII efficiency, and electron transfer efficiency. Previous studies have shown that the decrease in Fv/Fm under chilling stress may be caused by partial deactivation of the PSII reaction center [27]. A study by Han et al. [31] on chlorophyll biosynthesis in rice under low-temperature chilling stress found that the decrease in Fv/Fm at low temperatures might be due to the effects of low temperatures on the assembly and formation of PSII. Moreover, the increase in non-photochemical quenching (NPQ) at low temperatures suggests that PSII has low activity and needs to dissipate more excess light energy. In this study, chilling stress led to a significant decrease in Fv/Fm of maize leaves, indicating that the potential activity of PSII in leaves was inhibited and the photosynthetic electron transformation ability was weakened. Notably, Fv/Fm of the cold-tolerant variety was significantly higher than that of the conventional variety, indicating that the cold-tolerant variety had higher electron transfer ability and could still maintain high-efficiency photochemical reactions under chilling stress. The ability to recover photosynthetic function after cold removal is critical. For instance, the starch phosphorylase ZmPHOH has been identified as a key enzyme promoting photosynthetic recovery by regulating soluble sugar metabolism post-chilling [32]. This resilience of PSII is increasingly understood to depend on the stability of key thylakoid membrane protein complexes and the efficiency of the PSII repair cycle, both of which are targets of cold acclimation [33].

### 3.5. Antioxidant Defense and the Strong Association of POD Activity with Stress Tolerance

Abiotic stress often disrupts metabolic homeostasis, leading to an imbalance between the generation and scavenging of reactive oxygen species (ROS) and resulting in the accumulation of ROS in specific cellular compartments [34]. If excess light energy cannot dissipate in the form of heat, low-temperature-induced light inhibition also increases the risk of producing reactive oxygen species (ROS). Beyond physiological adaptations, the molecular basis of chilling tolerance involves complex regulatory networks. Transcriptomic and miRNA sequencing studies reveal that chilling stress rapidly alters the expression of numerous genes and microRNAs involved in antioxidant defense, hormone signaling, and developmental regulation, with distinct patterns between tolerant and sensitive genotypes [2]. Under chilling stress, SOD, POD, CAT, and other components of the antioxidant enzyme system often show decreased activity. It should be noted that our assay measured total SOD activity in leaf extracts. We acknowledge that this measure has limitations, as it may not reflect the dynamic and potentially opposite changes in specific SOD isoforms located in different subcellular compartments (e.g., Cu/Zn-SOD in cytosol/chloroplasts, Mn-SOD in mitochondria), which could be crucial for local ROS management and stress signaling. The relative contribution of distinct antioxidant components, including specific SOD isoforms and ascorbate peroxidase (APX), to chilling tolerance warrants further investigation at the subcellular level. Zhang et al. [6] found that chilling stress led to a sharp decline in SOD activity in maize, and the downtrend of SOD activity in cold-tolerant varieties was smaller than that in cold-sensitive varieties. In this study, the cold-tolerant variety showed higher antioxidant enzyme activity under chilling stress; its CAT and POD activities were significantly higher than those of the conventional variety, indicating that the cold-tolerant variety had a stronger antioxidant protection enzyme system. The results also showed that MDA content was significantly negatively correlated with activity of POD, CAT, and Pro content in maize leaves under chilling stress, and the correlation coefficient between MDA content and POD activity was the highest one among them, indicating that POD activity exhibited the strongest negative correlation with membrane damage (MDA) among the measured antioxidant enzymes under our experimental conditions. This suggests that POD activity could be a particularly prominent biomarker for the efficiency of the enzymatic antioxidant response in maize under delayed chilling stress. We recognize that a comprehensive understanding of the dominant regulatory mechanisms likely involves upstream factors such as local hormonal balance and intercellular coordination [35], which were not the focus of this physiological study. This aligns with research on sweet corn, where tolerant genotypes maintained higher activities of POD and SOD alongside lower MDA levels during low-temperature germination [3]. This aligns with the contemporary view that a coordinated “ROS-antioxidant interface,” rather than single enzyme activity, determines stress outcomes, with Class III peroxidases like POD playing a central role in fine-tuning H_2_O_2_ signaling and scavenging under cold stress [36]. Furthermore, the integration of antioxidant defense with osmotic adjustment (e.g., Pro accumulation) is now recognized as a synergistic mechanism that stabilizes membranes and proteins, a conceptual framework supported by recent multi-omics studies in cold-stressed crops [37]. While our study focused on GSH, future investigations should also consider the role of ascorbate (AsA) metabolism, a crucial component of photosynthetic electron transport and the chloroplastic ROS-scavenging network, in maize chilling tolerance.

## 4. Materials and Methods

### 4.1. Experimental Site

A two-year field experiment (2017 and 2018) was conducted under an irrigated cropping system at the research farm of Inner Mongolia Agricultural University (IMAU) in Tumote Youqi Banner County, Inner Mongolia, China (40°33′ N, 110°31′ E). The site represents a typical northwest China maize production region with a semi-arid continental monsoon climate. Total precipitation during the growing seasons was 324.8 mm (2017) and 442.2 mm (2018). Active accumulated temperature (≥10 °C) was 3083.7 °C·d (2017) and 2952 °C·d (2018).

The experimental field had sandy loam soil texture with continuous maize (*Zea mays* L.) cultivation. Composite soil samples (0–30 cm depth) were analyzed before land preparation. The study site consisted of sandy loam with the following initial properties: 20.5 g kg^−1^ organic matter, 105.84 mg kg^−1^ available nitrogen, 5.08 mg kg^−1^ available phosphorus, 161.93 mg kg^−1^ available potassium, and a pH of 7.4.

### 4.2. Experimental Design

A cold-tolerant maize variety (XY335, Tieling Pioneer Seed Research Co., Ltd., Tieling, China) and a cold-sensitive variety (KH8, Bayannur Kehe Seed Industry Co., Ltd., Bayannur, China) were subjected to germination tests under low and normal temperatures. Subsequently, both varieties were field-tested under two sowing dates simulating different chilling stress intensities. The delayed chilling stress treatment (CS) was sown 10 days earlier than the normal on-time sowing (OT). CS sowing dates were 16 April 2017 and 15 April 2018; OT dates were 26 April 2017 and 25 April 2018.

The experiment followed a randomized complete block design with three replications. Plots were 5 m long with 10 rows spaced 0.6 m apart. Plant density was 75,000 plants ha^−1^. Starter fertilizer (105 kg ha^−1^ P_2_O_5_, 40 kg ha^−1^ N [Diammonium phosphate, 46% P_2_O_5_, 18% N], and 45 kg ha^−1^ K_2_O [Potassium sulfate, 50% K_2_O]) was incorporated into the 0–15 cm soil layer before planting. At the 6-leaf stage, an additional 200 kg ha^−1^ N (Urea, 46% N) was side-dressed. Weeds were controlled using pre-plant and post-emergence herbicides. Flood irrigation supplied 50 mm of water at V10 and another 50 mm at R1 growth stages. Pesticides were applied via unmanned aerial vehicle as needed.

### 4.3. Sampling Procedures

**Germination:** Seed germination was tested using the paper towel method. Twenty-five seeds per variety (XY335, KH8) were placed on moistened paper towels (5 × 5 arrangement), covered with another towel, rolled, and stored in a growth chamber. Tests were conducted under normal temperature (NT, 25 °C) and low temperature (LT, 7 °C). For NT, germination potential was recorded on day 4; germination percentage and coleoptile length on day 7. For LT, germination potential was recorded on day 4; germination percentage and coleoptile length on day 14. Germination index (GI) and seed vigor index (SVI) were calculated as

GI = ΣGt/Dt (1)

where Gt is the number of seeds germinated on day t, and Dt is the corresponding germination day.

SVI = GI × Coleoptile length (2)

**Emergence:** Within a selected 3 m double row, seedling counts were recorded every 2 days starting at emergence onset. Physiological plant heights of 10 seedlings within the same section were measured at 15 days after emergence (DAE). The emergence index (EI) and seedling uniformity (SU) were calculated as [3]

EI = ΣGt/Dt (3)

where Gt is the number of seedlings emerged on day t, and Dt is the corresponding emergence day [3].

SU = Average plant height/Standard deviation (STDEV) of plant height (4)

**Leaf growth and root morphology:** After emergence, five fixed plants per plot were monitored for leaf growth. Leaf age was recorded every two days to calculate leaf growth rate from the onset of chilling stress (<15 °C). Post-stress, roots from three plants per treatment were excavated (0–30 cm soil depth), washed, and analyzed for root length, surface area, volume, and diameter using a WinRHIZO root scanning system (Pro, Regent Instruments Inc., Quebec, QC, Canada). The onset of chilling stress for CS plots was defined as the period beginning at emergence when the average daily soil temperature (at 5 cm depth) fell below 15 °C, as recorded by the on-site meteorological station.

**Biomass and yield:** Three consecutive plants per block were cut at the stem base at V6, R1, and R6 stages, oven-dried, and weighed for biomass determination. At physiological maturity (R6), ears from the central two rows per plot were hand-harvested. Kernel number per ear was recorded before shelling. Grain moisture, total biomass, and grain weight were measured. Grain yield was adjusted to 14% moisture content (140 g kg^−1^).

**Photosynthetic characteristics:** An LI-6400XT photosynthetic system (Li-Cor Inc., Lincoln, NE, USA) measured net photosynthetic rate (Pn), intercellular CO_2_ concentration (Ci), and stomatal conductance (Gs) on functional leaves at 2, 4, 6, 8, and 10 days after chilling stress onset. Maximum quantum efficiency of PSII (Fv/Fm) was measured using a HANDY PEA plant efficiency instrument (Hansatech Instruments Ltd., King’s Lynn, UK), and SPAD (chlorophyll index) using a SPAD-502Plus portable chlorophyll meter (Konica Minolta Sensing, Inc., Osaka, Japan). All measurements were taken on clear days between 09:00 and 11:30 local time. During measurements, the photosynthetic photon flux density (PPFD) was maintained at 1500 µmol m^−2^ s^−1^ using the LI-6400XT’s internal red-blue LED light source. Leaf chamber temperature was set to 25 °C, CO_2_ concentration to 400 µmol mol^−1^, and flow rate to 500 µmol s^−1^. For Fv/Fm measurements, leaves were dark-adapted for 30 min using leaf clips prior to measurement.

**Membrane stability and defense enzyme activity:** Total superoxide dismutase (SOD) activity was measured in leaf homogenates, representing the combined activity of SOD isoforms present in different subcellular compartments. Five days after chilling stress onset, malondialdehyde (MDA), soluble protein (Pro), superoxide dismutase (SOD), peroxidase (POD), catalase (CAT), and reduced glutathione (GSH) were measured according to Li [9].

**Assessment of chilling stress grade:** The method of Hou [1] was modified. Temperature data was sourced from the on-site meteorological station. Maize development stages were recorded. The relative active accumulated temperature anomaly (T~XJ~) for each period was calculated:

T_XJ_ = (ΣTi − ΣTOi)/ΣTO × 100% (5)

where ΣTi is the measured active accumulated temperature (°C·d) during development period I, ΣTOi is the standard active accumulated temperature (°C·d) for period I, and ΣTO is the total standard active accumulated temperature (°C·d) needed from emergence to maturity. Chilling stress occurrence and grade were determined by comparing T~XJ~ to standard thresholds for the corresponding variety and growth period [1].

### 4.4. Data Analysis

Data were analyzed using SPSS Statistics 27.0 (SPSS Inc., Chicago, IL, USA). Least significant difference (LSD) tests within the general linear model were performed at a *p* < 0.05 significance level. Linear regression and figure generation used SigmaPlot 12.5 (Systat Software Inc., San Jose, CA, USA).

## 5. Conclusions

This study elucidated the key physiological adaptations enabling cold-tolerant maize (XY335) to acclimate to delayed chilling stress:

(1) Root Morphology Plasticity: Shortening root length to maintain root diameter and absorption activity, ensuring shoot dry matter accumulation.
(2) Photosynthetic Stability: Maintaining higher chlorophyll content, PSII efficiency (Fv/Fm), and photosynthetic carbon assimilation capacity, supported by stable carbon/nitrogen metabolism.
(3) Growth Strategy: Employing a “standby mode” during stress followed by compensatory leaf growth acceleration upon temperature recovery.
(4) Robust Antioxidant Defense: Possessing a highly active antioxidant system, in which POD activity showed the strongest negative correlation with oxidative damage (MDA content), thereby effectively mitigating membrane lipid peroxidation.

These integrated physiological adaptations enable cold-tolerant maize varieties to achieve higher emergence, seedling vigor, and ultimately, stable yield under delayed chilling stress conditions.

## Figures and Tables

**Table 1:** Relative active accumulated temperature index (TXJ, %) during different maize growth stages under delayed chilling stress (CS) compared to chilling stress grade thresholds [1].

Year	Treatment	Emergence → 7-Leaf	Tasseling	Silking	Maturity
2017	CS	−2.1	39	42.2	94.7
2018	CS	−2.5	35.4	38.8	89.9
Chilling stress grade [1]	Slight	−1.5~−2.0	−1.6~−2.4	−1.8~−2.5	−3~−4.3
	Severe	<−2.0	<−2.4	<−2.5	<−4

Note: A negative TXJ value indicates a temperature deficit (chilling stress) relative to standard requirements for that growth period.

**Table 2:** Germination characteristics of maize varieties under normal (NT, 25 °C) and low temperature (LT, 7 °C) stress.

Treatment	Variety	Germination Percent (%)	Germination Index	Germination Potential (%)	Coleoptile Length (mm)	Seed Vigor Index
NT	XY335	85.5 a	7.6 a	72.7 a	13.6 a	103.4 a
	KH8	85.1 a	6.3 a	54.9 a	13.1 a	82.8 a
LT	XY335	66.1 a	2.5 b	0.0 b	3.7 b	9.2 b
	KH8	27.0 b	1.1 b	0.0 b	4.3 b	4.6 b
Difference (NT-LT)	XY335	19.4	5.1	72.7	9.9	94.2
	KH8	58.1	5.2	54.9	8.8	78.2

Note: Values within a column and treatment group (NT or LT) followed by different letters are significantly different (LSD, *p* < 0.05).

**Table 3:** Seedling emergence traits of maize varieties under normal (OT) and chilling stress (CS) treatments.

Year	Treatment	Variety	Emergence Percentage (%)	Emergence Index	Seedling Uniformity
2017	OT	XY335	99.4 a	14.3 a	76.6 a
		KH8	96.3 a	13.3 a	50.8 b
	CS	XY335	96.3 a	9.9 b	42.5 c
		KH8	83.3 b	8.7 b	12.6 c
	Difference (OT-CS)	XY335	3.1	4.4	34.1
		KH8	13.0	4.6	38.2
2018	OT	XY335	97.8 a	8.9 a	75.0 a
		KH8	97.8 a	8.0 a	42.7 b
	CS	XY335	92.0 b	5.7 b	39.3 b
		KH8	82.7 c	4.4 c	9.0 c
	Difference (OT-CS)	XY335	5.8	3.2	35.8
		KH8	15.1	3.6	33.7
Average	Difference	XY335	4.5	3.8	35.0
		KH8	14.1	4.1	36.0

Note: Values within a column and year followed by different letters are significantly different (LSD, *p* < 0.05).

**Table 4:** Yield and biomass accumulation of different maize varieties under normal (OT) and chilling stress (CS) treatments.

Year	Treatment	Variety	Biomass at R1 Stage (g plant^−1^)	Yield Components			Yield (t ha^−1^)	Yield Per Plant (g plant^−1^)
				Ears (10^4^ ha^−1^)	Kernel Number Per Ear	1000-Kernel Weight (g)		
2017	OT	XY335	151.6 a	7.2 a	584.2 a	359.9 a	12.8 a	178.4 a
		KH8	133.1 b	6.6 b	588.9 a	326.0 ab	10.7 b	163.1 b
	CS	XY335	141.7 a	7.0 a	585.5 a	347.0 ab	12.0 a	172.0 a
		KH8	116.3 c	6.1 c	589.4 a	301.1 b	9.2 b	150.8 b
	Difference (OT-CS)	XY335	9.9	0.2	−1.3	12.9	0.8	6.4
		KH8	16.8	0.5	−0.5	24.9	1.5	12.3
2018	OT	XY335	156.1 a	7.3 a	634.3 a	423.8 a	16.2 a	220.3 a
		KH8	146.8 b	6.9 ab	605.4 b	404.0 a	14.3 b	205.4 a
	CS	XY335	142.3 b	7.2 a	597.6 b	413.3 a	15.4 ab	196.5 a
		KH8	127.7 c	6.6 b	591.9 b	392.2 a	13.0 b	169.5 b
	Difference (OT-CS)	XY335	13.8	0.1	36.7	10.5	0.8	23.8
		KH8	19.1	0.3	13.5	11.8	1.3	36.0

Note: Values within a column and year followed by different letters are significantly different (LSD, *p* < 0.05).

## Data Availability

The original contributions presented in this study are included in the article. Further inquiries can be directed to the corresponding author.

## References

[1] Sommer M.L., Zhou Y., Hochholdinger F. (2025). Differential cold stress intensities drive unique morphological and transcriptomic changes in *Zea mays* root hairs. BMC Genom..

[2] Božić M., Ignjatović Micić D., Delić N., Nikolić A. (2024). Maize miRNAs and their putative target genes involved in chilling stress response in 5-Day old seedlings. BMC Genom..

[3] Wang T., Wu Z., Chen J., Li F., Lv G. (2024). The effects of chilling on antioxidant enzyme system and related gene expression levels in sweet corn seeds with different germination characteristics. Agric. For. Fish..

[4] Yan M., Li F., Sun Q., Zhao J., Ma Y. (2023). Identification of chilling-tolerant genes in maize via bulked segregant analysis sequencing. Environ. Exp. Bot..

[5] Ma Y., Yao L., Zhang L., Su A., Wang R., Song W., Li Z., Zhao J. (2023). Genome-wide association analysis of chilling-tolerant germination in a new maize association mapping panel. Food Energy Secur..

[6] Zhang Z.W. (2016). Identification of Maize Cold Tolerance and Its Regulation Effect. M.D. Dissertation.

[7] Li B., Fang Z.J. (2018). Screening of low-temperature tolerance maize inbred lines and analysis of leaf physiological characteristics and cellular structure changes. J. Henan Agric. Sci..

[8] He R.Y., Zheng J.J., Chen Y., Pan Z.Y., Yang T., Zhou Y., Li X.F., Nan X., Li Y.Z., Cheng M.J. (2023). QTL-seq and transcriptomic integrative analyses reveal two positively regulated genes that control the low-temperature germination ability of MTP–maize introgression lines. Theor. Appl. Genet..

[9] Lu Y., Li Y., Peng Q., Sun X., Yang Q., Song Z., Tian F., Yan Y., Liu M. (2025). Enhancing maize seed resistance to chilling stress through seed germination and surface morphological changes using high voltage electrostatic field. Sci. Rep..

[10] Zhang Y. (2013). Alleviative Effects and Its Mechanism of Exogenous Spermidine on Tomato Seedlings Under Salinity-Alkalinity Mixed Stress. Ph.D. Thesis.

[11] Wang S.W., Ma S.Q., Chen L., Wang Q., Huang J.B. (2009). Chilling Damage.

[12] Hund A., Richner W., Soldati A., Fracheboud Y., Stamp P. (2007). Root morphology and photosynthetic performance of maize inbred lines at low temperature. Eur. J. Agron..

[13] Farooq M., Aziz T., Wahid A., Lee D., Siddique K.H.M. (2009). Chilling tolerance in maize: Agronomic and physiological approaches. Crop Pasture Sci..

[14] Chen S., Long L., Sun X., Parsons D., Zhou Z. (2025). Responsive root traits and mitigating strategies for wheat production under single or combined abiotic stress. Eur. J. Agron..

[15] Shomo Z.D., Li F., Smith C.N., Edmonds S.R., Roston R.L. (2024). From sensing to acclimation: The role of membrane lipid remodeling in plant responses to low temperatures. Plant Physiol..

[16] Chen X.Y., Liu P., Cheng Y., Dong S., Zhang J.W., Zhao B., Ren B.C. (2019). Effects of phosphorus fertilizer application depths on root distribution and phosphorus uptake and utilization efficiencies of summer maize under subsoiling tillage. Acta Agron. Sin..

[17] Giauffret C., Bonhomme R., Derieux M. (1995). Genotypic differences for temperature response of leaf appearance rate and leaf elongation rate in field-grown maize. Agronomie.

[18] Blacklow W.M. (1972). Influence of temperature on germination and elongation of the radicle and shoot of corn (*Zea mays* L.). Crop Sci..

[19] Riva-Roveda L., Escobar B., Giauffret C., Perilleux C. (2016). Maize plants can enter a standby mode to cope with chilling stress. BMC Plant Biol..

[20] Lainé C.M.S., AbdElgawad H., Beemster G.T.S. (2024). Cellular dynamics in the maize leaf growth zone during recovery from chilling depends on the leaf developmental stage. Plant Cell Rep..

[21] Ogawa A., Yamauchi A. (2007). Root osmotic adjustment under osmotic stress in maize seedlings 2. Mode of accumulation of several solutes for osmotic adjustment in the root. Plant Prod. Sci..

[22] Theocharis A., Clément C., Barka E.A. (2012). Physiological and molecular changes in plants grown at low temperatures. Planta.

[23] Bilska A., Sowinski P. (2010). Closure of plasmodesmata in maize (*Zea mays*) at low temperature: A new mechanism for inhibition of photosynthesis. Ann. Bot..

[24] Liu Y.F., Qi M.F., Li T.L. (2012). Photosynthesis, photoinhibition and antioxidant system in tomato leaves stressed by low night temperature and their subsequent recovery. Plant Sci..

[25] Zhang G., Liu Y., Ni Y., Meng Z., Lu T., Li T. (2014). Exogenous calcium alleviates low night temperature stress on the photosynthetic apparatus of tomato leaves. PLoS ONE.

[26] Farquhar G.D., Sharkey T.D. (1982). Stomatal conductance and photosynthesis. Annu. Rev. Plant Physiol..

[27] Kao W., Shih C., Tsai T. (2004). Sensitivity to chilling temperatures and distribution differ in the mangrove species *Kandelia candel* and *Avicennia marina*. Tree Physiol..

[28] Yang M.S., Wang Y.F., Gan X.X., Luo H., Zhang Y., Zhang W. (2012). Effects of exogenous nitric oxide on growth, antioxidant system and photosynthetic characteristics in seedling of cotton cultivar under chilling injury stress. Sci. Agric. Sin..

[29] Han M., Cao B.L., Liu S.S., Xu K. (2018). Effects of rootstock and scion interaction on photosynthesis and nitrogen Metabolism of grafted tomato seedlings leaves under low temperature stress. Acta Hortic. Sin..

[30] Leipner J. (2009). Chilling Stress in Maize from Physiology to Genetics and Molecular Mechanisms. Ph.D. Thesis.

[31] Han Q.H. (2017). Effect of Low Temperature Stress on Chlorophyll Biosynthesis and Chloroplast Development of Rice (*Oryza sativa* L.) Seedlings. M.D. Dissertation.

[32] Qin Y., Ding H., Zhao H., Zheng X., Wang J., Xiao Z., Wang Y., Wang H., Liu Y., Gong D. (2025). A starch phosphorylase, ZmPHOH, improves photosynthetic recovery from short-term cold exposure in maize. Int. J. Mol. Sci..

[33] Gu J., Liu P., Nie W., Wang Z., Cui X., Fu H., Wang F., Qi M., Sun Z., Li T. (2025). Abscisic acid alleviates photosynthetic damage in the tomato ABA-deficient mutant sitiens and protects photosystem II from damage via the WRKY22–PsbA complex under low-temperature stress. J. Integr. Agric..

[34] You D.L. (2016). Effect of Exogenous Spermidine on Physiology and Polyamine Metabolism of Maize (*Zea mays* L.) Seedlings Under Waterlogging Stress. M.D. Dissertation.

[35] Ma Y., Tan R., Zhao J. (2022). Chilling Tolerance in Maize: Insights into Advances—Toward Physio-Biochemical Responses’ and QTL/Genes’ Identification. Plants.

[36] Singh A., Satheeshkumar P.K., Mishra A.K. (2024). Reactive oxygen species (ROS) and ROS scavengers in plant abiotic stress response. Stress Biology in Photosynthetic Organisms.

[37] Liu M., Liu M., Wu X., Li M., Li S., Xiong T., Li C., Li C., Tang Y., Tang Y. (2025). Multi-omics analysis reveals the physiological and molecular response to cold stress in different spring wheat cultivars at the booting stage. Front. Plant Sci..

